# Psychological Resilience of Chinese College Students: A Cross-Sectional Study after the Deblocking of China’s COVID-19 Pandemic Strategy

**DOI:** 10.3390/healthcare11172409

**Published:** 2023-08-28

**Authors:** Rong Zhao, Jin Peng, Jia-Yin Li, Lu-Lu Qin, Bang-An Luo

**Affiliations:** 1 Brain Hospital of Hunan Province (The Second People’s Hospital of Hunan Province), Changsha 410007, China; 2 School of Medicine, Hunan Normal University, Changsha 410013, China

**Keywords:** psychological resilience, family function, self-efficacy, mental health, college students

## Abstract

Psychological resilience (PR) plays an important role in fortifying mental health during pandemics. This study aimed to examine the PR and its related factors of college students in China after the deblocking of the China’s COVID-19 pandemic strategy. A total of 1100 college students from 15 universities participated in this cross-sectional survey by multi-stage stratified sampling. Data were collected by self-designed socio-demographic information, the family function assessment scale (APGAR), a general health questionnaire (GHQ-12), the general self-efficacy scale (GSES), and a psychological resilience scale. The average score of PR was 135.65 ± 18.54. Cluster analysis of PR scores showed that 24.9% of the college students had weak PR. The higher PR, the higher mental health status (r = 0.352, *p* < 0.05). Females had higher PR than males (OR = 0.550, 95% CI: 0.367–0.827). High self-efficacy was an independent protective factor of high PR (OR = 0.093, 95% CI: 0.059–0.145). Low family contact frequency, poor family function, and bad mental health status were the independent risk factors of high PR. In conclusion, the PR of Chinese college students were insufficient after the deblocking of China’s COVID-19 pandemic strategy, indicating an improvement of PR should be put into practice immediately. Frequent monthly contact with family, family function, self-efficacy, mental health status, and gender were the influencing factors of PR, which provides an intervention strategy for the future.

## 1. Introduction

Psychological resilience (PR) is a multidimensional concept, which refers to “the relative resistance to environmental risk experience or the overcoming of adversity” [1], such as a pandemic. PR highlights the good adaptation of individuals after experiencing major difficulties or trauma. The process of forming PR can be roughly divided into three steps: (1) individuals experience adversity or stress, and discover the protective factors of PR or the quality of overcoming adversity; (2) individuals possess these PR qualities or protective factors through their own integration; and (3) individuals gain experience and recover from interference or adversity [2]. Studies have shown that the PR varied from each of the individuals [3,4]. In addition, the level of individual PR is not fixed [1]. On one hand, although individual PR can be cultivated through some steps, different individuals face different risk environments; on the other hand, the individual makes different responses when facing risks at different ages and in different environments. A study showed that the PR is the core element of well-being [5], which is very important to prevent and alleviate mental health problems [6]. At the same time, PR is regarded as one of the key abilities of higher educational graduates in the 21st century [7].

During the COVID-19 global health pandemic, calls have been made to address the associated public health crisis, including public mental health. Since December 2019, China’s epidemic prevention and control program used isolation at home and mask wearing as the core methods to reduce the spread of the virus between persons [8,9]. The measure of isolation at home caused great changes in the lives of college students [10]. During the 3-year-lockdown period in China, many studies have suggested that the rates of anxiety and depression during the epidemic were higher than in ordinary conditions [11,12,13,14,15].

Based on the ability of coping with adversity [16], PR has been discussed in previous studies. From the perspective of positive psychology, higher PR helps students get rid of negative emotions faster and adapt better [17]. Firstly, some previous studies on college students’ PR mostly studied the relationship between single factors and PR. For example, military training may play a positive role in improving Freshmen’s PR and reducing depressive symptoms; the scores of patients’ Health Questionnaire-9 (PHQ-9) decreased significantly [18]. Secondly, many researchers study the PR of college students focused on their different characteristics. For example, some researchers pointed out that college students diagnosed with ADHD (Attention-deficit hyperactivity disorder) may represent a particularly resilient group [19]. Additionally, other studies take PR as an intermediate factor to explore whether it has an impact and the size of its impact. For instance, during the COVID-19 pandemic, a study on the impact of college students’ stress and anxiety on their sleep showed that the enhancement of PR weakens the relationship between perceived stress and anxiety regarding sleep quality [20].

On the 7th of December 2022, China announced it would end its 3-year COVID-19 lockdown strategy. Although the above research has provided important clues in the mechanism of PR, to date, few studies have looked specifically at the mechanism of the PR of college students as the outcome variable, especially after the deblocking of China’s COVID-19 pandemic strategy. 

On the 3rd of January 2023, the World Health Organization’s (WHO’s) Director General concurred with the advice offered by the Committee regarding the ongoing COVID-19 pandemic and determined that the event continues to constitute a public health emergency of international concern (PHEIC) [21]. Thus, considering the dynamic process of PR, it is a serious and important affair to understand the situation of the PR among the college students after the deblocking of China’s COVID-19 pandemic strategy, indicating that there is a need to study this phenomenon. Meanwhile, in order to understand PR fully, based on previous studies, we intend to investigate more factors related to PR such as family, general self-efficacy, and mental health status [16,17,18,19,20]. 

While evidence about the effects of PR on COVID-19 is emerging, the trajectories of PR remain unknown, especially after the deblocking of China’s COVID-19 pandemic strategy. Identifying more influencing factors may contribute to better PR and sharing experiences and information, which is of utmost importance. Therefore, since PR is a protective factor of mental health, from the perspective of positive psychology this study aims to explore PR and its related factors among Chinese college students after the deblocking of China’s COVID-19 pandemic strategy, by considering the role that PR has played in bolstering mental health status during the COVID-19 pandemic, and in building positive processes and capacities that may help individuals cope with and grow through the pandemic. 

## 2. Materials and Methods

### 2.1. Study Population and Procedures

This was a cross-sectional study conducted in five regions (Beijing, Guangxi, Hunan, Hainan, and Xizang) of China in late December 2022, when China announced the deblocking of its COVID-19 pandemic strategy. Sample size was calculated using the formula for cross-sectional studies: α = 0.05, n = u_α/2_^2^P(1 − P)/d^2^ (“P” refers to the estimated proportion and “d” stands for the error), where u = 1.96 when α = 0.05. By the multi-stage stratified cluster sampling, a total of 1100 college students from 15 universities participated in the questionnaire survey, with an efficiency rate of 98.0% (1078/1100). 

During this survey, the investigation is both through a paper form and electronic form of the questionnaire. In the Hunan province, we used the paper form of the questionnaire for students who were willing to participate in this study, the paper form questionnaires were distributed and retrieved by our trained investigators; for other districts, the electronic form of the questionnaire was preferred, which was emailed to participants by our trained investigators.

### 2.2. Data Collection and Measurements

#### 2.2.1. Socio-Demographic Information

This study uses a self-designed questionnaire to collect the socio-demographic information of participants, including gender, age, nationality, family residence, grade, family economic level, average monthly living expenses in school, monthly contact frequency with family, the education level of parents, and parents’ marital status.

#### 2.2.2. Family Function Survey

The Adaptation-Partnership-Growth-Affection-Resolve Questionnaire (APGAR), compiled by Smilkstein of the University of Washington, is mainly used to evaluate an individual’s family function, including five items: adaptability, cooperation, growth, emotion, and intimacy [22]. Each question has 3 options, of which “rarely” to “often” are scored 0–2 points, respectively. The total score is 0–3, indicating a serious impairment of family function, 4–6 indicating a moderate impairment of family function, and 7–10 indicating a good family function. The higher the score, the better the family function. Studies have shown that the questionnaire has good reliability and validity, and has good internal consistency reliability in the Chinese population (Cronbach’s α = 0.91) [23]. 

#### 2.2.3. Mental Health Status

The 12-item general health questionnaire (GHQ-12) was used to evaluate the mental health status of the respondents in recent weeks [24]. GHQ-12 can be widely used in the epidemiological investigation of mental disorders and the screening of mental disorders in the community population. At the same time, it can be used as a reliable and effective tool to measure the mental health status of Chinese college students. According to the scoring method recommended by the WHO, the GHQ-12 uses a binary scoring method, which means that 0 or 1 point is calculated for each question. The positive responses non pathologic (e.g., “feeling good”, “as usual”) are scored as 1, and the negative pathologic responses (e.g., “feeling bad”, ”negative evaluation”) are scored as 0. The total score is within the range of 0–12 points. The higher the score, the lower the level of mental health. The higher the score, the greater the possibility of mental disorder. Studies at home and abroad generally believe that the best cut-off value of GHQ-12 score is 3 points. GHQ screening positive means that the total score is higher than or equal to 3 points, and negative means the total score is less than 3 points. Evidence shows that GHQ-12 has good reliability and validity in the Chinese population, and the Cronbach’s α is 0.75 [25]. 

#### 2.2.4. General Self-Efficacy

The general self-efficacy scale (GSES), compiled by German professor Ralf Schwarzer, is mainly used to evaluate self-efficacy [26]. The scale adopts Likert’s 4-point scoring (1 = completely incorrect, 4 = completely correct), and the total score is 10–40 points. The higher the total score, the higher the self-efficacy of the subjects. Some scholars have shown that the scale has good reliability and validity, and has good internal consistency reliability among college students in China (Cronbach’s α = 0.92) [27].

#### 2.2.5. Psychological Resilience

In this study, the Resilience Scale of College Students compiled by Liu Lanlan of Hebei Normal University was used to evaluate the PR of the respondents [28]. The scale is composed of eight dimensions: social communication ability, family support, friend support, optimistic and tolerant attitude, self-worth, self recognition, self-control, and self adjustment. It includes 38 items, including 8 reverse scoring items: 5-level scoring, 1 point for complete nonconformity, 2 points for comparative nonconformity, 3 points for uncertainty, 4 points for comparative conformity, and 5 points for complete conformity. Reverse scoring items are scored forward. The higher the score, the better the state of resilience. The total scale has good reliability and validity (Cronbach’s α = 0.91). However, because the Resilience Scale used in this study does not provide a reference boundary value of resilience classification, this study combines the resilience classification and hierarchical cluster analysis results obtained by previous researchers [9,28,29], and takes the psychological resilience score as the clustering index for k-means cluster analysis. The samples are divided into three categories: strong resilience, intermediate resilience, and weak resilience.

### 2.3. Statistical Analysis

The statistical software SPSS 24.0 (IBM Corp., Armonk, NY, USA) was used to conduct statistical analysis. Descriptive analyses were analyzed by mean ± standard deviation ($\bar{\chi }$ ± s, the mean is represented by $\bar{\chi }$, the standard deviation is represented by s), frequency, and rate. The χ^2^ test and *t* test were used to compare the differences of PR status among college students with different characteristics. The Mantel-Haenszel χ^2^ (ordered categorical form variables) and the Pearson Correlation Coefficient (continuous form variables) were performed to conduct the correlation analyses. The ordered logistic regression analysis was conducted to explore the influencing factors of PR among college students in China with the odds ratio (OR) and its 95% confidence interval (CI). The test level α is 0.05.

## 3. Results

### 3.1. The Characteristics of the Study Population

Among the respondents, men accounted for 35.1% (378/1078) and women accounted for 64.9% (700/1078). The average age of the participants was 20.26 (±1.25) years. Participants of Han nationality accounted for 87.6% (944/1078); 56.8% (612/1078) of them lived in urban areas; 91.7% (989/1078) of their parents were in a stable marriage, whose degrees of education were mainly junior high school or high school; 61.0% (658/1078) of the participants had a medium family economic level; 78.3% (844/1078) of the respondents had an average monthly living expense of more than 1000 yuan/month; 56.6% (610/1078) of them had a good family function; and 59.6% (642/1078) of them were in contact with family more than 5 times a month.

### 3.2. Psychological Resilience of College Students in China

The average score of PR of college students in China was (135.65 ± 18.543), which varied from 70 to 188. Among them, 268 (24.9%) had low PR, 516 (47.9%) had intermediate PR, and 294 (27.3%) had high PR. Among the eight dimensions of PR, “family support” scored the highest (26.77 ± 4.662), while “self-control” scored the lowest (10.27 ± 2.211). (See Table 1 for specific results).

### 3.3. Family Function, Mental Health Status, and Self-Efficacy of College Students in China

Among the 1078 respondents, the average score of the family function survey was 6.99 ± 2.572, the highest score was 10 and the lowest was 0. Among them, 112 had severe family dysfunction, 356 had moderate family dysfunction, and 610 had good family function, accounting for 10.4%, 33.0%, and 56.6%, respectively. The average score for individuals with severe family function disorders is 2.29 ± 0.889, the average score for individuals with moderate family function disorders is 5.13 ± 0.732, and the average score for individuals with good family function is 8.93 ± 1.168. Among the five dimensions, “degree of cooperation” scored the lowest (1.22 ± 0.739) and “degree of growth” scored the highest (1.58 ± 0.588). See Table 2 for detailed results (Table 2).

The average score of mental health status was 2.16 ± 2.377, with a highest score of 12 and a lowest score of 0. In the mental health status survey, there were 324 positive respondents and 754 negative respondents, accounting for 30.1% and 69.9%, respectively. The average score of positive respondents and negative respondents was 5.17 ± 2.125 and 0.87 ± 0.753, respectively (Table 3).

The average score of the self-efficacy survey was 25.01 ± 5.375, with a highest score of 40 and a lowest score of 12. See Table 3 for specific results.

### 3.4. Distribution of Psychological Resilience among Different Characteristics of College Students

Results showed that females have higher PR scores than males (137.13 ± 18.177, 95% CI: 135.26~139.19 vs. 132.89 ± 18.943, 95% CI: 130.40~135.44; t = 2.544, *p* < 0.05). Additionally, the distribution of PR among students with different gender, family economic status, monthly contact frequency with family, family function, and mental health status were significant (*p* < 0.05). The results are shown in Table 4 and Table 5. 

### 3.5. Correlations Analysis of Psychological Resilience 

Results showed that the PR was positive, which significantly correlated with family function and self-efficacy, however, the PR was negative, which significantly correlated with family contact frequency, mental health, and family economic level (*p* < 0.01) (Table 6).

### 3.6. Influencing Factors of PR among College Students in China

Taking PR as the dependent variable, the ordered logistic regression analysis method was conducted. Gender, family economic status, monthly contact frequency with family, family function, mental health, father’s education level, mother’s education level, parents’ marital status, and monthly living expenses were taken as independent variables, and the scores of self-efficacy were used as a covariate for regression analysis. Results showed that monthly contact frequency with family, family function, self-efficacy score, mental health status, and gender were the influencing factors of PR. The independent protective factors for the reduction of high PR are being a woman (OR = 0.550, 95% CI = (0.367, 0.827)) and a high sense of efficacy (OR = 0.093, 95% CI = (0.059, 0.145)). The independent risk factors of high PR were low family contact frequency, poor family function, and unhealthy mental health. The PR of college students with contact 2–4 times per month with their family (OR = 1.600, 95% CI = (1.078, 2.377)) or contact frequency ≤1 time (OR = 3.053, 95% CI = (1.041, 8.953)), moderate impairment of family function (OR = 3.114, 95% CI = (2.024, 4.797)) or severe family dysfunction (OR= 11.858, 95% CI = (5.680, 24.730)), and unhealthy mental state (OR = 3.633, 95% CI = (2.237, 5.906)) was low. Additionally, considering the gender, we conducted the stratified analysis according to gender. The results of stratified analysis according to gender showed that the PR of males was impacted by independent variables such as monthly contact with family, family function, mental health and self-efficacy. However, the PR of females was impacted by independent variables such as family function, mental health, and self-efficacy. See Table 7 and Table 8 for detailed results.

## 4. Discussion

### 4.1. The PR of College Students in China after the Deblocking of China’s COVID-19 Pandemic 

After the deblocking of China’s COVID-19 pandemic strategy, only 27.3% of Chinese college students had high PR during COVID-19, while 24.9% of the students had low PR, indicating that the PR of Chinese college students should be improved. Compared to previous studies during the COVID-19 pandemic in China, the PR level of college students after the deblocking of China’s COVID-19 pandemic strategy were similar to that reported in such studies [9,29]. Zeng et al. [9] investigated an independent college in Guangdong province in 2020 by using the Psychological Resilience Scale proposed by Hu and Gan (five-point Likert scale), the score of PR was 3.417 ± 0.443; and in our previous investigation conducted in the Hunan province in 2021 by using the Chinese version of the Resilience Scale for College Students (RSCS), constructed by Liu, the PR of college students was 137.97 ± 15.50, which is close to the median, indicating that the participants’ PR was at a medium level [29]. However, the PRs of this study were lower than students in some other countries. Sarmiento et al. reported that 57.7% of the university students in Spain presented high resilience [30]; Surzykiewicz et al. found that the resilience in the Polish population during the COVID-19 period were 18.64 ± 5.28 (ranging from 0–30) [31]. 

PR is varied in different regions, populations, and individuals. As previous studies reported, the framework of PR has both internal and external factors [32], and adversity is an important factor in the concept of PR [33]. Internal factors of PR include biological and psychological factors, such as gender, physical condition, intelligence, and so on. External factors include family, society, countries, peer group, isolation measures, etc. However, the stress and adversity are obviously non-randomly distributed among individuals [1]. So, the enhancement of PR lies not in avoiding stress, but in how people deal with life changes and how they act in the face of adversity [34]. 

### 4.2. Influencing Factors of PR among Chinese College Students after the Deblocking of China’s COVID-19 Pandemic Strategy

According to the Coping with COVID-19 with Resilience (COPERS) study initiated by the international Public Mental Health Section of the European Public Health Association (EUPHA), it is important to identify the PR trajectories of adults aged 18 years and older and to detect factors that potentially influence their PR in the different countries [35]. The premise of improving college students’ PR is to understand the protective or risk factors related to PR. In this study, these factors are mainly personal factors and family environmental factors, which is consistent with previous research. The results of ordered logistic regression analysis showed that the independent protective factors for the reduction of high PR include a high sense of efficacy, and the independent risk factors of high PR were weak self-confidence, low frequency of family contact, poor family function, and unhealthy mental health, and females are likely to have higher PR than males.

(1) PR and self-efficacy

There was a positive correlation between self-efficacy and PR among Chinese college students after the deblocking of China’s COVID-19 pandemic strategy. The stronger the individual’s self-efficacy, the higher the level of PR, which is consistent with other research [36,37,38,39,40,41]. As previous studies reported, the correlation between PR and self-efficacy is quite high. For example, if individuals do not have a sense of self-efficacy, they will lack sufficient motivation to persist in difficulties or adversity, and it will be difficult to produce resilience [38]. In a study on nurses’ academic burnout, it is pointed out that there is a significant relationship between PR and self-efficacy, and students with a higher PR score higher in academic efficacy. The higher the general self-efficacy of college students, the more likely they are to adopt optimistic and positive thinking and behavior in coping with adversity [40]. Some researchers explained the reasons for the above conclusions as follows: self-efficacy is an important factor of PR, and a high level of self-efficacy can be used as a stress buffer to improve PR [41].

(2) PR and mental health

After the deblocking of China’s COVID-19 pandemic strategy, the PR of Chinese college students is positively correlated with mental health, which indicates a predictive effect of resilience to mental health. The higher the level of resilience, the better the state of mental health. A domestic study on whether college students have left behind experience points out that mental health status problems are negatively correlated with resilience [42]. Foreign scholars have also pointed out that resilience is negatively correlated with mental health status problems such as pain, depression, anxiety, unhappiness, and other domains [43,44,45]. In adversity, compared with young people with lower resilience, young people with higher resilience are less likely to have mental health status problems and can better cope with adversity [46]. Thus, some frustration with education and training could be added to the work of mental health status promotion among students.

(3) PR and monthly contact with family and family function

Related to the COVID-19 pandemic, this is the first study to give evidence of the two factors (monthly contact with family, family function) having a positive impact on the PR. The family provides a key support for individuals: the more actively parents encourage, the more they can make their children focus on their career goals [47]; the warmer family members felt, the easier it is to help individuals cultivate confidence in overcoming difficulties and the ability to adapt to the environment [48]; and the existence of a family connection provides protection for the students [34], such as financial assistance, advice when children are confused, etc. [49]. The reason for this phenomenon may be related to the children’s attachment to their parents. The higher the degree of parent–child attachment, the lower the level of social interaction anxiety and the higher the psychological resilience [50].

(4) PR and gender

After the deblocking of China’s COVID-19 pandemic strategy, results from this study show that the PR score of females was higher than that of males, while research of medical students showed that the PR score of female medical students was lower than that of male medical students [51]. As for the opposite results, Rutter once explained that females seem to be less vulnerable than males when facing psychosocial adversity, and women are less likely to cause destructive behavior in others [34]. However, Hirani and Hegadoren hold a different opinion and gave a detailed explanation, they pointed out that although women’s scores on resilience indicators are generally lower than those of men, the existing concept of resilience does not reflect gender roles and differently shapes the experiences of women and men and their responses to adversity [52]. And, according to the results of the stratified analysis by gender in this study, the PR of females and males were independently affected by monthly contact with family, family function, mental health, and self-efficacy. However, for female students, the PR of females was independently affected by family function, mental health and self-efficacy. Only the factor of monthly contact with family was different among female and male students, which might be caused by the contact habit differences between females and males. Therefore, more research about the resilience among different genders should be explored.

Basing on the above findings, consistent with previous studies [30,31], socio-demographic variables have no significant way to build resilience during the COVID-19 pandemic. However, this study found that the importance of family support (monthly contact with family and family function) as a protective factor against anxiety, loneliness, fear, and other negative feelings experienced during college life during the COVID-19 pandemic. Therefore, schools and families should pay more attention to family support for students. Situations similar to the COVID-19 pandemic may occur in the future, and intervention strategies should be put in place to meet the family support required. Additionally, some intervention measures should be put in practice to tackle the absence of family support. More research is needed to study effective ways to improve and maintain a good psychological resilience.

However, there are some limitations to this study and our results should be interpreted with caution. First of all, this study is limited by its cross-sectional analysis, thus the causal relationships cannot be obtained. Secondly, all data are from the questionnaire survey, which is easily affected by the mood and living environment of the subjects during this investigation, so the data can only reflect the feelings of the respondents in the period before the survey. Thirdly, the respondent biases were unavoidable as a result of the self-reported design. Finally, this survey was only conducted in China, which limits its generalization to other areas.

## 5. Conclusions

After the deblocking of China’s COVID-19 pandemic strategy, the PR of Chinese college students were insufficient. Monthly contact with family, family function, self-efficacy, mental health status, and gender may provide more intervention strategies for future.

## Figures and Tables

**Table 1:** Scores of psychological resilience.

Variables	Score ($\bar{\chi }$ ± s)	95% CI
Total PR	135.65 ± 18.543	134.21~137.19
High PR (n = 294, 27.3%)	158.08 ± 9.814	156.50~159.78
Intermediate PR (n = 516, 47.9%)	135.16 ± 6.217	134.42~135.87
Low PR (n = 268, 24.9%)	111.96 ± 9.407	110.28~113.52
Eight dimensions of PR		
(1) Social communication competence	21.74 ± 4.857	21.35~22.20
(2) Family support	26.77 ± 4.662	26.36~27.18
(3) Friend support	23.33 ± 3.558	23.02~23.61
(4) Attitude of optimism and tolerance	14.57 ± 2.541	14.36~14.79
(5) Self-value	14.23 ± 2.994	13.97~14.50
(6) Self-recognition	14.27 ± 2.619	14.05~14.48
(7) Self-control	10.27 ± 2.211	10.09~10.47
(8) Self-adjustment	10.47 ± 1.921	10.31~10.63

**Table 2:** Score of family function of college students in China.

Variables	Score ($\bar{\chi }$ ± s)	95% CI
Family function	6.99 ± 2.572	6.77~7.21
Severe family dysfunction (n = 112, 10.4%)	2.29 ± 0.889	2.04~2.50
Moderate impairment of family function (n = 356, 33.0%)	5.13 ± 0.732	5.03~5.25
Good family function (n = 610, 56.6%)	8.93 ± 1.168	8.79~9.07
Five dimension of family function		
(1) Adaptability	1.33 ± 0.692	1.27~1.39
(2) Cooperation	1.22 ± 0.739	1.15~1.28
(3) Growth	1.58 ± 0.588	1.52~1.63
(4) Emotion	1.30 ± 0.670	1.24~1.36
(5) Intimacy	1.56 ± 0.576	1.51~1.61

**Table 3:** Scores of mental health status and self-efficacy of Chinese college students.

Variables	Score ($\bar{\chi }$ ± s)	95% CI
Mental health status	2.16 ± 2.377	1.96~2.37
Negative status (n = 754, 69.9%)	5.17 ± 2.125	4.87~5.47
Positive status (n = 324, 30.1%)	0.87 ± 0.753	0.79~0.94
Self-efficacy status	25.01 ± 5.375	24.57~25.50

**Table 4:** Comparison of psychological resilience score among gender.

Gender	Psychological Resilience Score		*t*	*p*
	$\bar{\chi }$ ± s	95% CI		
Male	132.89 ± 18.943	(130.40, 135.44)	2.544	0.011
Female	137.13 ± 18.177	(135.26, 139.19)		

**Table 5:** Distribution of PR among different characteristic college students (N = 1078).

Variables	N (%)	PR			χ^2^	*p*
		Weak	Intermediate	Strong		
Gender					15.520	<0.001 ^#^
Male	378 (35.1%)	120	170	88		
Female	700 (64.9%)	148	346	206		
Age(year)					11.337	0.183
≤18	106 (9.8%)	20	52	34		
19	122 (11.3%)	29	61	32		
20	376 (34.9%)	113	175	88		
21	358 (33.2%)	82	170	106		
≥22	116 (10.8%)	24	58	34		
Nation					0.126	0.949
Han nationality	944 (87.6%)	236	452	256		
Other ethnic groups	134 (12.4%)	32	64	38		
Home residence					5.819	0.055
Rural	466 (43.2%)	125	231	110		
Urban	612 (56.8%)	143	289	184		
Marriage status of parents *					4.206	0.125
Stable	989 (91.7%)	238	477	274		
Unstable	89 (8.3%)	30	39	20		
Grade					14.635	0.067
Freshman	130 (12.1%)	24	62	44		
Sophomore	64 (5.9%)	20	34	10		
Junior	480 (44.5%)	134	222	124		
Senior	380 (35.3%)	84	184	112		
Five-grade student	24 (2.2%)	6	14	4		
Mother’s educational level					12.888	0.116
Junior high school below	228 (21.4%)	68	112	48		
Junior high school	352 (32.7%)	90	174	88		
High school	275 (25.5%)	64	128	83		
College	196 (18.2%)	40	90	66		
Graduate student or above	27 (2.5%)	4	12	9		
Father’s educational level					13.162	0.106
Junior high school below	170 (15.8%)	48	81	41		
Junior high school	328 (30.4%)	87	156	85		
High school	305 (28.3%)	86	136	83		
College	248 (22.4%)	42	125	74		
Graduate student or above	34 (3.2%)	5	18	11		
Family economic level					28.722	<0.001 ^#^
Below medium	324 (30.1%)	110	152	62		
Medium	658 (61.0%)	134	324	200		
Above medium	96 (8.9%)	24	40	32		
Average monthly living expenses (yuan/month)					7.875	0.087
<500	8 (0.7%)	2	4	2		
500–1000	226 (21.0%)	72	93	61		
>1000	844 (78.3%)	196	417	231		
Monthly contact frequency with family					60.419	<0.001 ^#^
<1 time/month	40 (3.7%)	20	16	4		
2–4 times/month	396 (36.7%)	110	222	64		
>5 times/month	642 (59.6%)	138	278	226		
Family function					230.067	<0.001 ^#^
Severe	112 (10.4%)	74	30	8		
Moderate	356 (33.0%)	120	196	40		
Good	610 (56.6%)	74	290	246		
Mental health status					72.395	<0.001 ^#^
Negative status	754 (69.9%)	213	392	149		
Positive status	324 (30.1%)	55	124	145		

* Stable marital status: refers to married and remarried; unstable marital status: refers to divorced, widowed, unmarried, and other marital status, the same below; ^#^ *p* < 0.05.

**Table 6:** Correlations of PR with other variables.

Variables	Mantel-Haenszel χ^2^	Pearson Correlation Coefficient
Monthly contact frequency with family	21.821 **	−0.202 **
Mental health status	66.853 **	−0.353 **
Family economic level	9.750 **	−0.135 **
Family function	99.493 **	0.430 **
Self-efficacy		0.458 **

** *p* < 0.01.

**Table 7:** Ordered logistic analysis of PR of college students in China.

Variables	B	SE	Walds	*p*	OR	95% CI of OR	
						Lower	Upper
Gender							
Male					1.000		
Female	−0.597	0.208	8.258	0.004	0.550	0.367	0.827
Monthly contact frequency with family (times/month)							
>5					1.000		
2–4	0.470	0.202	5.425	0.020	1.600	1.078	2.377
<1	1.116	0.549	4.129	0.042	3.053	1.041	8.953
Family function							
Good					1.000		
Moderate	1.136	0.220	26.634	0.000	3.114	2.024	4.797
Severe	2.473	0.375	43.431	0.000	11.858	5.680	24.730
Mental health status							
Positive					1.000		
Negative	1.290	0.248	27.118	0.000	3.633	2.237	5.906
Self-efficacy score	−2.377	0.228	108.655	0.000	0.093	0.059	0.145

**Table 8:** Ordered logistic analysis of PR of college students in China by gender.

Variables	Female		Male	
	OR	95% CI	OR	95% CI
Monthly contact frequency with family (times/month)				
>5	1.000		1.000	
2–4	2.514	1.434~3.410	3.353	2.413~5.042
<1	5.529	4.154~8.265	7.941	6.281~10.426
Family function				
Good	1.000		1.000	
Moderate	3.016	2.390~4.811	3.700	2.659~6.273
Severe	8.053	6.813~10.985	5.936	3.234~9.796
Mental health status				
Positive	1.000		1.000	
Negative	7.471	5.385~10.637	2.489	1234~4.590
Self-efficacy score	0.089	0.031~0.167	0.108	0.019~0.216

## Data Availability

Data are available from the Department of Mental Health, Brain Hospital of Hunan Province of China (contact via hnjswszx@126.com) for researchers who meet the criteria for access to confidential data.

## References

[1] Rutter M. (1993). Resilience: Some conceptual considerations. J. Adolesc. Health Off. Publ. Soc. Adolesc. Med..

[2] Richardson G.E. (2002). The metatheory of resilience and resiliency. J. Clin. Psychol..

[3] Tusaie K., Puskar K., Sereika S.M. (2007). A predictive and moderating model of psychosocial resilience in adolescents. J. Nurs. Scholarsh..

[4] Tusaie K., Dyer J. (2004). Resilience: A historical review of the construct. Holist. Nurs. Pract..

[5] Stoffel J.M., Cain J. (2018). Review of Grit and Resilience Literature within Health Professions Education. Am. J. Pharm. Educ..

[6] Amanda F.S., Dahlberg E.E., Thompson S.C. (2018). Systematic review of resilience-enhancing, universal, primary school-based mental health promotion programs. BMC Psychol..

[7] Tomlinson M. (2017). Introduction: Graduate Employability in Context: Charting a Complex, Contested and Multi-Faceted Policy and Research Field.

[8] Kang L., Li Y., Hu S., Chen M., Yang C., Yang B., Wang Y., Hu J., Lai J., Ma X. (2020). The mental health of medical workers in Wuhan, China dealing with the 2019 novel coronavirus. Lancet Psychiatry.

[9] Zeng W., Wu X., Xu Y., Wu J., Zeng Y., Shao J., Huang D., Zhu Z. (2021). The Impact of General Self-Efficacy on Psychological Resilience During the COVID-19 Pandemic: The Mediating Role of Posttraumatic Growth and the Moderating Role of Deliberate Rumination. Front. Psychol..

[10] Wang C., Pan R., Wan X., Tan Y., Xu L., Ho C.S., Ho R.C. (2020). Immediate Psychological Responses and Associated Factors during the Initial Stage of the 2019 Coronavirus Disease (COVID-19) Epidemic among the General Population in China. Int. J. Environ. Res. Public Health.

[11] Chang J., Yuan Y., Wang D. (2020). Mental health status and its influencing factors among college students during the epidemic of COVID-19. J. South. Med. Univ..

[12] Tang W., Hu T., Hu B., Jin C., Wang G., Xie C., Chen S., Xu J. (2020). Prevalence and correlates of PTSD and depressive symptoms one month after the outbreak of the COVID-19 epidemic in a sample of home-quarantined Chinese university students. J. Affect. Disord..

[13] Cao W., Fang Z., Hou G., Han M., Xu X., Dong J., Zheng J. (2020). The psychological impact of the COVID-19 epidemic on college students in China. Psychiatry Res..

[14] Gan Y., Ma J., Wu J., Chen Y., Zhu H., Hall B.J. (2020). Immediate and delayed psychological effects of province-wide lockdown and personal quarantine during the COVID-19 outbreak in China. Psychol. Med..

[15] Yang X., Jia W., Xia G., Wang J., Yu W. (2015). Analysis of the influence of social hierarchy background on depression and anxiety among college students. Chin. J. Dis. Control Prev..

[16] Xu H., Su C., Xu Y., Li Y., Jie Y., Ma X., Zhou H., Yang Y., Liu Q. (2018). Analysis of reciprocity between mental health status and academic achievement under the protective effect of psychological resilience in junior school students in earthquake-hit area. J. Hyg. Res..

[17] Yi F., Li X., Song X., Zhu L. (2020). The Underlying Mechanisms of Psychological Resilience on Emotional Experience: Attention-Bias or Emotion Disengagement. Front. Psychol..

[18] Guo R., Sun M., Zhang C., Fan Z., Tao H. (2021). The Role of Military Training in Improving Psychological Resilience and Reducing Depression Among College Freshmen. Front. Psychiatry.

[19] Wilmshurst L., Peele M., Wilmshurst L. (2011). Resilience and Well-being in College Students with and without a Diagnosis of ADHD. J. Atten. Disord..

[20] Du C., Zan M.C.H., Cho M.J. (2020). Increased Resilience Weakens the Relationship between Perceived Stress and Anxiety on Sleep Quality: A Moderated Mediation Analysis of Higher Education Students from 7 Countries. Clocks Sleep.

[21] https://www.who.int/.

[22] Smilkstein G. (1978). The family APGAR: A proposal for a family function test and its use by physicians. J. Fam. Pract..

[23] Feng Y., Fan Y., Su Z., Li B., Li B., Liu N., Wang P. (2021). Correlation of Sexual Behavior Change, Family Function, and Male-Female Intimacy Among Adults Aged 18–44 Years During COVID-19 Epidemic. Sex. Med..

[24] Xin Y., Li Z. (2022). COVID-19 Worry and Mental Health Among the Economically Active Population in Guangdong, China. Front. Public Health.

[25] Yang T.Z., Huang L., Wu Z.Y. (2003). The application of Chinese health questionnaire for mental disorder screening in community settings in mainland China. Chin. J. Epidemiol..

[26] Scholz U., Doña B., Sud S., Schwarzer R., Rica C. (2002). Is general self-efficacy a universal construct? Psychometric findings from 25 countries. Eur. J. Psychol. Assess..

[27] Zhou C., Yue X.D., Zhang X., Feng S., Zhang X.Y. (2021). Self-efficacy and mental health problems during COVID-19 pandemic: A multiple mediation model based on health belief model. Personal. Individ. Differ..

[28] Liu L. (2007). The Development and Application in the Resilience Scale of University Students.

[29] Qin L.L., Peng J., Shu M.L., Liao X.Y., Gong H.J., Luo B.A., Chen Y.W. (2023). The Fully Mediating Role of Psychological Resilience between Self-Efficacy and Mental Health: Evidence from the Study of College Students during the COVID-19 Pandemic. Healthcare.

[30] Serrano Sarmiento Á., Sanz Ponce R., González Bertolín A. (2021). Resilience and COVID-19. An Analysis in University Students during Confinement. Educ. Sci..

[31] Surzykiewicz J., Konaszewski K., Skalski S., Dobrakowski P.P., Muszyńska J. (2021). Resilience and Mental Health in the Polish Population during the COVID-19 Lockdown: A Mediation Analysis. J. Clin. Med..

[32] Mandleco B.L., Peery J.C. (2000). An organizational framework for conceptualizing resilience in children. J. Child Adolesc. Psychiatr. Nurs..

[33] Luecken L.J., Gress J.L., Reich J., Zautra A., Hall J. (2009). Early adversity and resilience in emerging adulthood. Handbook of Adult Resilience.

[34] Rutter M. (1985). Resilience in the face of adversity. Protective factors and resistance to psychiatric disorder. Br. J. Psychiatry.

[35] Backhaus I., Sisenop F., Begotaraj E., Cachia J., Capolongo S., Carta M.G., Jakubauskiene M., Jevtic M., Nakov V., Pirlog M.C. (2021). Resilience and Coping With COVID-19: The COPERS Study. Int. J. Public Health.

[36] Di Corrado D., Muzii B., Magnano P., Coco M., La Paglia R., Maldonato N.M. (2022). The Moderated Mediating Effect of Hope, Self-Efficacy and Resilience in the Relationship between Post-Traumatic Growth and Mental Health during the COVID-19 Pandemic. Healthcare.

[37] Wha L.T., Kyung K.Y. (2010). Effects of self-efficacy, affectivity and collective efficacy on nursing performance of hospital nurses. J. Adv. Nurs..

[38] Bandura A. (2011). A Social Cognitive perspective on Positive Psychology. Rev. De Psicol. Soc..

[39] Thomas L.J., Revell S.H. (2016). Resilience in nursing students: An integrative review. Nurse Educ. Today.

[40] García-Izquierdo M., Ríos-Risquez M.I., Carrillo-García C., Sabuco-Tebar E. (2018). The moderating role of resilience in the relationship between academic burnout and the perception of psychological health in nursing students. Educ. Psychol..

[41] Katja S., Jessica F., Lena D., Laura H.A., Eike S., Michèle W. (2021). Psychological Network Analysis of General Self-Efficacy in High vs. Low Resilient Functioning Healthy Adults. Front. Psychiatry.

[42] Shi J., Chen Z., Yin F., Zhao J., Zhao X., Yao Y. (2016). Resilience as moderator of the relationship between left-behind experience and mental health of Chinese adolescents. Int. J. Soc. Psychiatry.

[43] Haddadi P., Besharat M.A. (2010). Resilience, vulnerability and mental health. Procedia-Soc. Behav. Sci..

[44] Mei S.T.L., Ni A.O.Z., Sivaguru S.A., Cong C.W. (2021). Social support, resilience, and happiness in response to COVID-19. J. Cogn. Sci. Hum. Dev..

[45] Corral-Verdugo V., Corral-Frías N.S., Frías-Armenta M., Lucas M.Y., Peña-Torres E.F. (2021). Positive environments and precautionary behaviors during the COVID-19 outbreak. Front. Psychol..

[46] Peng L., Zhang J., Li M., Li P., Zhang Y., Zuo X., Miao Y., Xu Y. (2012). Negative life events and of Chinese medical students: The effect of resilience, personality and social support. Psychiatry Res..

[47] Crombie A., Brindley J., Harris D., Marks-Maran D., Thompson T.M. (2013). Factors that enhance rates of completion: What makes students stay?. Nurse Educ. Today.

[48] Masten A.S., Garmezy N. (1985). Risk, Vulnerability, and Protective Factors in Developmental Psychopathology.

[49] Murrel A.R., Ryeshia J., Teresa H. (2018). Psychological Flexibility and Resilience in Parentally Bereaved College Students. Omega.

[50] Yu Y., Liu S., Song M., Fan H., Zhang L. (2020). Effect of Parent–Child Attachment on College Students’ Social Anxiety: A Moderated Mediation Model. Psychol. Rep..

[51] Adam N., Greg M. (2019). Exploring the relationship between medical student basic psychological need satisfaction, resilience, and well-being: A quantitative study. BMC Med. Educ..

[52] Hirani S., Lasiuk G., Hegadoren K. (2016). The intersection of gender and resilience. J. Psychiatr. Ment. Health Nurs..

